# Oncogenic *KRAS*-Induced Feedback Inflammatory Signaling in Pancreatic Cancer: An Overview and New Therapeutic Opportunities

**DOI:** 10.3390/cancers13215481

**Published:** 2021-10-31

**Authors:** Sapana Bansod, Paarth B. Dodhiawala, Kian-Huat Lim

**Affiliations:** 1 Division of Oncology, Department of Internal Medicine, Barnes-Jewish Hospital and The Alvin J. Siteman Comprehensive Cancer Center, Washington University School of Medicine, St. Louis, MO 63110, USA; bansod@wustl.edu (S.B.); dodhi001@umn.edu (P.B.D.); 2 Medical Scientist Training Program, University of Minnesota Medical School, Minneapolis, MN 55455, USA

**Keywords:** inflammation, stroma, pancreatic cancer, IRAK4, TPL2, TAK1, MK2

## Abstract

**Simple Summary:**

Oncogenic KRAS signaling drives several effector cascades that contribute not only to the malignant behavior of pancreatic cancer cells but also formation of the fibro-inflammatory microenvironment. The non-neoplastic cells in the tumor microenvironment interact with tumor cells, creating a complex onco-inflammatory signaling network that enhances the resilience of cancer cells and potentially explaining why targeting KRAS effectors is clinically ineffective. We provide a focused review of this onco-inflammatory network and discuss novel therapeutic opportunities that can be developed.

**Abstract:**

Pancreatic ductal adenocarcinoma (PDAC) remains highly refractory to treatment. While the *KRAS* oncogene is present in almost all PDAC cases and accounts for many of the malignant feats of PDAC, targeting KRAS or its canonical, direct effector cascades remains unsuccessful in patients. The recalcitrant nature of PDAC is also heavily influenced by its highly fibro-inflammatory tumor microenvironment (TME), which comprises an acellular extracellular matrix and various types of non-neoplastic cells including fibroblasts, immune cells, and adipocytes, underscoring the critical need to delineate the bidirectional signaling interplay between PDAC cells and the TME in order to develop novel therapeutic strategies. The impact of tumor-cell KRAS signaling on various cell types in the TME has been well covered by several reviews. In this article, we critically reviewed evidence, including work from our group, on how the feedback inflammatory signals from the TME impact and synergize with oncogenic KRAS signaling in PDAC cells, ultimately augmenting their malignant behavior. We discussed past and ongoing clinical trials that target key inflammatory pathways in PDAC and highlight lessons to be learned from outcomes. Lastly, we provided our perspective on the future of developing therapeutic strategies for PDAC through understanding the breadth and complexity of KRAS and the inflammatory signaling network.

## 1. Introduction

Pancreatic ductal adenocarcinoma (PDAC) is projected to be the second leading cause of cancer deaths in the USA by 2030 [1]. Currently, there is no reliable diagnostic tool to detect PDAC at an early stage to allow surgical intervention, which is the only known path for cure. Instead, ~90% of PDAC cases are diagnosed when the tumors have grown beyond the extent of surgery or metastasized, leaving systemic chemotherapy as the only treatment option. However, chemotherapies are rarely effective as single agents in PDAC. As such, combinatorial regimens such FOLFIRINOX (cocktail of oxaliplatin, irinotecan, leucovorin, and 5-fluorouracil) and gemcitabine plus nab-paclitaxel are needed to augment efficacy and achieve meaningful prolongation of survival [2,3]. Unfortunately, these regimens are not universally effective and have significant side effects. Other therapeutic modalities including targeted and immunotherapies, while showing much promise in preclinical studies and which are already standard treatment options in other cancer types, have remained largely unsuccessful for PDAC patients. Only a very small percentage (~0.5–0.8%) of PDAC cases possess microsatellite instability or high mutational burden which render them potentially responsive to checkpoint immunotherapy [4,5]. Due to these challenges, current 5-year overall survival of all PDAC patients remains low, at around 9% [6].

Although the genetic aberrations that underlie PDAC have been very well-established, therapeutic strategies targeting these events have not yet been successful in the clinic. The *KRAS*, *TP53*, *CDKN2A/B* and *SMAD4* genes are among the most commonly mutated genes in PDAC [7]. About 95% of PDAC tumors harbor a gain-of-function mutation of the *KRAS* gene [8,9], and the prognosis of these cases are significantly worse than those with wild-type *KRAS* [10], making it a bone-fide therapeutic target in PDAC. However, targeting KRAS has not been realized in PDAC. The recently developed clinically effective KRAS^G12C^-specific inhibitors have a very limited role in PDAC due to the extreme rarity (<1%) of G12C mutation in this disease. Effective KRAS^G12D^ and KRAS^G12V^ inhibitors are still in preclinical development [11,12]. Tremendous effort in the past few decades was focused on targeting the direct downstream effector cascades of KRAS including the RAF-MEK-ERK (MAPK), PI3K-AKT-mTOR and RalGDS-RalA/RalB pathways. Of these, the MAPK pathway is regarded as one of the most critical [13]. Unfortunately, neither RAF nor MEK inhibitors alone have shown clinical efficacy in PDAC patients [14,15,16]. Clinical trials combining MEK and PI3K/AKT inhibitors have also failed to show efficacy and are hindered by significant toxicities [17,18,19,20]. These setbacks underscore the critical need to comprehensively re-appraise the breadth of oncogenic feats driven by mutant KRAS for identifying novel therapeutic targets and developing combinatorial strategies that have a higher chance of success in clinical trials.

A salient feature that distinguishes PDAC from other *KRAS*-mutant cancers such as lung and colon cancers is its extensive fibro-inflammatory stroma, which typically accounts for 80–85% of the tumor bulk. The PDAC stroma is heavily infiltrated with various non-neoplastic cell types including the immune cells, fibroblasts, and vascular endothelial cells. Importantly, these cells are recruited and reprogrammed by PDAC cells during tumor initiation, driven largely by oncogenic KRAS signaling emanating from PDAC cells. Through direct physical interaction and secreted factors, these cells constantly communicate with PDAC cells, creating a dynamic bi-directional cell-to-cell communication that is linked with intrinsic KRAS signaling in PDAC cells. Therefore, the “ripple effect” of oncogenic KRAS, in the context of a complex multicellular TME, effectually extends beyond tumor-intrinsic effector cascades and encompasses feedback signaling from the surrounding cells, which are critical in shaping the recalcitrant phenotype of PDAC cells. For example, the extracellular matrix and stromal cells in the TME can promote epithelial-mesenchymal transition of PDAC cells [21]. The impact of oncogenic KRAS on the surrounding cell types, particularly fibroblasts, vascular, endothelial, and immune cells, was recently reviewed by Carvalho et al. [22], Hamrsheh et al. [23], Kitajima et al. [24], and Stone et al. [25]. These insightful reviews highlight the importance of disrupting the mechanism by which *KRAS*-mutant cancer cells subvert the surrounding cells to create a pathologic TME that fosters invasion, metastasis, and treatment resistance. However, a reviews that focuses on the feedback signaling from the non-neoplastic cells on the malignant behavior of PDAC cells are lacking. Here, we provided a critical appraisal of the impact of TME on different inflammatory pathways in PDAC cells. Because the cellular outcome of each inflammatory pathway is highly cell-type dependent, we focused only on literature that uses PDAC cells and animal models. We portrayed a PDAC-specific “onco-inflammatory” network and discussed novel therapeutic opportunities based on seminal literature and recent findings from our group. We discussed important past and recent clinical trials that target inflammatory pathways in PDAC and provided our perspectives on targeting inflammation in PDAC in future clinical trials.

## 2. Inflammation Propels *KRAS*-Induced Neoplastic Progression in PDAC

Inflammation is the most established environmental factor that propels KRAS-induced neoplastic progression in PDAC. In human patients, *KRAS* mutations are detected in >90% of early staged pre-cancerous pancreatic intraepithelial neoplasias (PanINs) [26], but transformation to full-blown cancer occurs at a very low incidence, and is more much likely in patients with chronic pancreatitis or obesity, conditions which are associated with chronic systemic inflammation [27,28,29]. This association is well recapitulated in genetically engineered mouse models (GEMMs), in which pancreas-specific expression of oncogenic *KRAS (KRAS^G12D^)* alone is highly inefficient in inducing PanINs or PDAC, predominantly due to oncogene-induced cellular senescence [30,31]. However, the senescence program can be overcome through induction of inflammation or concomitant ablation of tumor suppressor genes *TP53*, *CDKN2A/B*, *INK4a/ARF*, or *SMAD4* [5,7,32]. When treated with cerulean to induce chronic pancreatitis, adult mice expressing oncogenic *KRAS^G12D^* developed PanIN and PDAC at 100% and 30% incidence, respectively [30,33]. In another GEMM in which *KRAS^G12D^* expression can be reversibly switched on/off by doxycycline treatment, maintenance of neoplastic epithelia, activation of fibroblasts, and infiltration of immune cells require the continual expression of *KRAS^G12D^*, which upregulates different tumor-intrinsic signaling pathways including the sonic-Hedgehog and inflammatory IL-6/STAT3 pathways [34]. Genetic ablation of *STAT3* from *KRAS^G12D^*-expressing epithelial cells inhibits PanIN progression and reduces the development of PDAC [35,36]. Therefore, tumor-intrinsic inflammatory signaling not only overcomes the senescence barrier that hinders transformation of pre-malignant, *KRAS*-mutated cells, but is also a critical mechanism through which *KRAS*-mutant cells subvert the external stromal and immune cells.

## 3. Forward Circuitry: Oncogenic KRAS Drives Inflammatory Cytokines to Shape the TME

Inflammation is a host defense mechanism that is invoked secondarily to foreign insults including the microbes or foreign antigens. The inflammatory response is typically initiated by recognition of pathogen-associated molecular patterns (PAMPs) or damage-associated molecular patterns (PAMPs) by the Toll-like receptors (TLRs) on host cells. Cancer inflammation shares many of these features but is also distinct in a few ways. Through expressing cancer “neoantigens” as a result of gene mutations, cancer cells can be recognized as “foreign” entities, like microbes, by the host immune system, which is then engaged to eliminate or contain these cancer cells. However, cancer cells are completely distinct from microbes or lifeless foreign bodies in a several ways. First, cancer cells such as PDAC can actively secrete multiple different inflammatory cytokines or chemokines controlled by oncogene signaling. These cytokines and chemokines can heavily influence the composition of incoming immune cells and fibroblasts and subsequently the net immunologic outcome. Second, despite being phenotypically altered, cancer cells still retain many normal cellular functions including recognition of surrounding DAMPs and PAMPs and activation of innate inflammatory pathways to draw immune cells. Third, cytokines secreted by the immune cells or fibroblasts, which in the setting of infection have no effect on the invading microbes, have active and significant signaling function within cancer cells and thus can affect their behavior. Fourth, the feedback signaling impact from the external stimuli, including cytokines, chemokines, DAMPs, or PAMPs can interact with the intrinsic oncogenic pathways, and may aggravate cancer cell behavior. Moreover, cytokines/chemokines secreted by cancer cells, and the impact of the external stimuli on cancer cells are very different between cancer types and their underlying oncogenic mutations. Based on the exceedingly intense fibro-inflammatory histology of PDAC compared with other cancer types, we posit such “onco-inflammatory” circuitry is particularly vigorous in PDAC.

**Figure 1:**
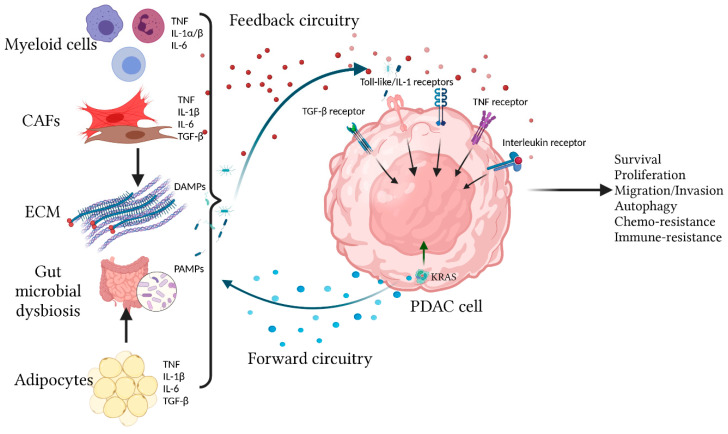
Overview of the bi-directional onco-inflammatory network between PDAC cells and surrounding cells. Oncogenic KRAS signaling in transformed PDAC cells drives effector cascades leading to secretion of inflammatory cytokines/chemokines such as IL-1α/β, IL-6, and CXCL1 to the TME. These secreted factors not only have autocrine signaling functions but also have paracrine effects by recruiting and reprogramming the surrounding non-neoplastic cells, causing them to secrete more cytokines/chemokines into the TME. These cytokines/chemokines provide additional signaling feedback to PDAC cells through multiple receptors including the TIR (Toll-like/IL-1) receptors, TNF, TGF-β, and Interleukin receptors (IL-6R being the best studied). These receptors utilize overlapping and distinct signal transduction mechanisms to affect cellular outcome, which include production of more cytokines/chemokines, proliferation, survival, migration, autophagy, and resistance to chemotherapy and immune surveillance.

Studies in isogenic human cell lines showed that expression of oncogenic *RAS* drives expression of multiple inflammatory cytokines/chemokines including IL-6, IL-8, CXCL1, CXCL2, and CXCL5 [37,38]. Notably, IL-6 not only has an autocrine role in supporting tumorigenesis in vivo, but also in a paracrine manner recruits vascular endothelial cells to support tumor angiogenesis [38]. In addition, studies from our group showed that oncogenic *KRAS* promotes secretion of IL-1β through the MEK-ERK cascade, leading to activation and migration of cancer-associated fibroblasts (CAFs) [39,40]. Importantly, the ability of PDAC cells to recruit the surrounding cells and reprogram the TME is mostly dependent on the intrinsic feature of the PDAC cells. In a *KRAS*-mutated murine PDAC cell model, tumor-intrinsic CXCL1 expression alone determines the quantitative and qualitative features of infiltrative CD8^+^ T cells and hence response to checkpoint immunotherapy [41]. In addition, after successfully metastasizing to a distant organ, PDAC cells establish a new TME that is remarkably similar to the primary tumor, featuring a highly fibrotic stroma, heavy infiltration of suppressive myeloid cells, and exclusion of cytotoxic T cells [42,43]. The intense desmoplastic histologic feature is not a cardinal feature of other *KRAS*-mutant cancer types such as non-small cell lung cancer (NSCLC), colorectal cancer, (CRC) or multiple myeloma. Much of this discrepancy is probably attributed to co-existing cancer-specific genetic or epigenetic aberrations of each cancer type, which can form different genetic and signaling networks with oncogenic KRAS [44]. Beyond the scope of cancer cells, it is entirely possible that the unique tissue-specific niche in which the *KRAS*-mutant cancer cells arise critically determines the behavior of these cancer cells, ultimately shaping the unique behavior of each cancer type. The differences in tumor-intrinsic (co-existing genetic/epigenetic aberrations) and -extrinsic (tumor niche) factors likely explain the discrepancy in the therapeutic outcome of targeting KRAS in different cancer types. For instance, KRAS^G12C^ inhibitor sotorasib had a 32% objective response rate in *KRAS^G12C^*-mutated NSCLC, but only 7% and 9% in *KRAS^G12C^*-mutated CRC and PDAC, respectively. The median duration of response was 10.9 months for NSCLC and 5.4 months for CRC [45]. Therefore, the final phenotypic output of oncogenic *KRAS* is highly tissue-specific. In the context of PDAC, oncogenic *KRAS* signaling communicates with the surrounding non-neoplastic cells via exchange of inflammatory chemokines and cytokines, which over time culminates in a bi-directional “onco-inflammatory” communication network that shapes the malignant behavior of PDAC tumors (Figure 1).

## 4. Feedback Circuitry: Inflammatory Signaling from the TME Activates KRAS-Dependent and Independent Pathways to Augment PDAC Cell Fitness

Compared with most other cancer types, PDAC is distinguished by an ultra-low neoplastic cellularity, which accounts for ~10–15% of tumor bulk, and a dense stroma that consists of an acellular extracellular matrix (ECM) infiltrated with CAFs and immune cells [46,47]. Therefore, it is highly plausible that the ECM and surrounding cells have a predominant role in signaling crosstalk with the PDAC cells at every stage of tumorigenesis including initiation, maintenance, and metastasis. As support, high dimensional single-cell RNAseq analysis shows that at every stage of neoplastic progression from early PanIN and late PanIN to PDAC, there are several transcriptomically distinct subpopulations of neoplastic cells, stromal fibroblasts, and immune cells that seem to co-evolve [48,49]. Importantly, these studies were performed in *KRAS^G12D^*-driven GEMM with defined genetic mutations, and thus the heterogeneous subpopulations of neoplastic cells actually reflect the impact of epigenetic or transcriptomic changes under the heavy influence from the TME. Similarly, single-cell RNAseq of PDAC CAFs revealed at least three distinct transcriptomic subtypes (myofibroblastic, inflammatory, and antigen-presenting CAFs) that are affected by their proximity to adjacent PDAC cells. Importantly these subtypes are interchangeable depending on the environmental clues and culture conditions [50,51]. While from these studies it is impossible and probably unnecessary to delineate the “chicken-and-egg” relationship between the neoplastic cells and surrounding non-neoplastic cells, it is certain that there is constant signaling crosstalk and ultimately a selection of symbiotic interaction between these two cell populations that permits progression from PanIN to PDAC.

**Figure 2:**
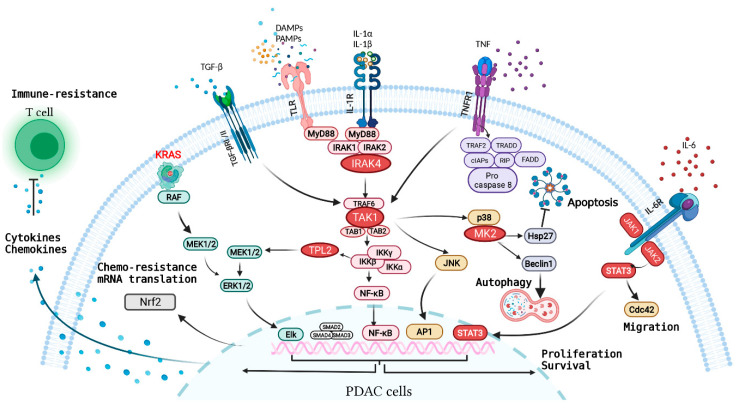
Overview of major inflammatory receptors and the associated signaling network in PDAC cells. Oncogenic KRAS in transformed PDAC cells drives secretion of inflammatory cytokines/chemokines such as IL-1α/β, IL-6, and CXCL1 that subvert other cell types in the TME, causing production of more cytokines and chemokines in the TME. These secreted factors engage with inflammatory receptors on PDAC cells, driving a network of signaling pathways that synergize with oncogenic KRAS signaling in propelling various malignant feats of PDAC.

In the following section, we summarize how the inflammatory clues from the TME affects signaling and behavior of PDAC cells. Due to the vast amount of literature in this area, we focus on four inflammatory pathways that are the most well-described and for which therapeutic strategies have been developed (Figure 2).

### 4.1. Toll-Like/Interleukin-1 Receptor (TIR) Pathway

The Toll-like/IL-1 Receptors (TIR) are a large family of receptors that share a highly conserved cytoplasmic domain and thus utilize overlapping downstream signaling mechanisms. Each of the TLR and IL-1R families consist of at least ten structurally related members [52,53]. These receptors are ubiquitously expressed in all cell types and function as the “first line responders” that initiate and sustain inflammation. In brief, these receptors are activated by interleukins (IL-1α/β, IL-18, IL-33, and IL-36 for the IL-1R family) as well as PAMPs or DAMPs (for the TLRs). Engagement of these receptor results in recruitment of distinct sets of adaptor proteins such as MyD88, TRIF, TRAM, and Mal, which then initiates a cascade of kinases including Interleukin-1 Receptor-associated kinases (IRAK), transforming growth factor-β-activated kinase 1 (TAK1), leading to activation of downstream NF-κB transcription factors p38/mitogen-activated protein kinase (MAPK) and the c-Jun N-terminal kinase (JNK) pathways. Outcomes from these events include enhanced cell proliferation and survival, as well as secretion of more pro-inflammatory cytokines and immunomodulatory molecules including the interferons and tumor-necrosis factors (TNFs). These factors are critical in attracting and activating CAFs, and innate and adaptive immune cells to collectively trigger local and systemic inflammatory responses [54].

#### 4.1.1. IL-1R Family Members

The IL-1R superfamily consists of various structurally-related members that can be triggered by a suite of cytokines including IL-1α, IL-1β, IL-18, IL-33, and IL-36 [55]. These ligands can be actively secreted by PDAC cells, CAFs, and immune cells, or released from necrotic cells as alarm cytokines. In a PDAC GEMM (*PDX-Cre; LSL-KRAS^G12D^; Ink4a/Arf^F/F^)*, oncogenic KRAS activates the AP-1 transcription factor, leading to enhanced transcription of IL-1α. In an autocrine manner, IL-1α promotes ubiquitination of tumor necrosis factor (TNF) receptor-associated factor 6 (TRAF6) at lysine 63 and results in the activation of IκB kinase-beta (IKKb) and the p62/NF-κB factors which subsequently transactivate IL-1α and p62. This study showed that the KRAS-IL-1α-p62 feedforward circuit is crucial for PDAC tumorigenesis and provides a strong rationale for therapeutically targeting IL-1R signaling to disrupt this circuit [56]. As opposed to IL-1α, which is synthesized in an active form, active IL-1β is produced through regulated proteolytic cleavage of inactive pro-IL-1β by the NLRP3 inflammasome and caspase 1 [57,58]. In PDAC cells, KRAS oncoprotein utilizes the MEK-ERK effector cascade to upregulate the transcription of *IL-1β* [39]. In an autocrine manner, IL-1β engages IRAK4 to constitutively activate the NF-kB and MEK-ERK cascades in PDAC cells, leading to enhanced invasiveness, production of various cytokines including IL-1α, IL-1β, IL-6, IL-8, CCL2, and CXCL1, as well as augmentation of PDAC cell survival and chemotherapy resistance [59,60,61]. In a paracrine manner, PDAC-secreted IL-1β recruits and activates CAFs, which also utilize IRAK4 to produce more type-1 collagen and IL-1β into the TME, forming a heterotypic feedforward inflammatory circuit that strengthens the viability of PDAC cells [40].

#### 4.1.2. Toll-Like Receptors (TLRs)

At least ten different TLRs have been identified in humans. Each TLR is specialized in recognizing distinct DAMPs or PAMPs such as lipopolysaccharides, DNAs, RNAs, flagellin, and DAMPs such as heat-shock proteins, hyaluronan, DNAs, and RNAs [53], all of which are abundant within PDAC TME. Several TLRs are overexpressed and contribute to the malignant behavior of PDAC cells. For instance, TLRs2, 4, and 9 are overexpressed in PDAC cells and control autocrine cell proliferation through expression of VEGF and PDGF [62]. Pancreatic adenocarcinoma upregulated factor (PAUF), a secreted protein that is overexpressed in PDAC, binds to TLR2 and TLR4 to activate the TPL2-MEK-ERK cascade and promote expression of RANTES and MIF [63]. Treatment with lipopolysaccharides (LPS), a ligand for TLR4, promotes PDAC cell invasion in a MyD88- and NF-kB-dependent manner [64]. Ligation of TLR7 and TLR8 results in elevated NF-kB and COX expression, which in turn promotes tumor growth and resistance to 5-fluorouracil chemotherapy [65]. Further, exposure of PDAC cells to chemotherapy such as irinotecan markedly induced TLR9 expression in an NF-kB dependent manner, leading to TLR9-dependent activation of IRAK4 and its downstream kinase TPL2. TPL2 then further enhances both the MEK-ERK and NF-kB pathway activation to augment resistance to chemotherapy-induced apoptosis [39]. In a *KRAS^G12D^*-driven GEMM, inflammatory stimuli such as cerulean trigger an NF-κB-mediated positive feedback mechanism involving coclyoxygenase-2 (Cox-2) that augments RAS activity to a pathologically high level, which is required to propel progression of pre-malignant cells to PDAC [66,67]. In these studies, the mechanism by which RAS-activity is augmented is unclear but likely results from ligation of autocrine growth factor and cytokine receptors, which can activate the wild-type isoforms and/or promote the GDP/GTP cycling of mutant KRAS.

Emerging evidence shows that gut microbiome, an abundant source of TLR ligands, has a critical role in inflammation and treatment resistance in PDAC [68]. A comprehensive study on 1526 PDAC tumors with matched adjacent normal tissues showed that PDAC tumors are dominated by *Proteobacteria* including the Enterobacteriales and Pseudomonadales species, which are naturally abundant in normal duodenum [69]. In support, another study showed that *Proteobacteria*, *Bacteroidetes*, and *Firmicutes* were the most abundant microbes in human PDAC samples [70]. In PDAC GEMMs, transplantation of fecal material from tumor-bearing *PDX-Cre;LSL-KRAS^G12D^;TP53^R172H^* (KPC) mice suppresses both the innate and adaptive immunity and accelerates PDAC development in the *PDX-Cre;LSL-KRAS^G12D^* (KC) mice, which on their own have very low incidence in developing PDAC. Conversely, depletion of the gut microbiome in KPC mice with antibiotics leads to a diminution of infiltrative suppressive myeloid cells infiltration and reprogramming of TAMs toward a tumor-protective M1-like phenotype [70]. Mechanistically these microbes induce expression of multiple TLRs on both PDAC cells and macrophages, leading to tumor cell proliferation and immune suppression. Besides bacteria, fungal mycobiome, specifically the *Malassezia* spp., is also enriched in the PDAC samples of human and KPC mice. Through binding to mannose-binding lectin (MBL) and activating the complement cascade, *Malassezia* spp. promotes *KRAS^G12D^*-driven PDAC progression in KC mice. Elimination of gut fungal mycobiome with a potent antifungal agent amphotericin-B promotes the efficacy of gemcitabine [71]. Together, these studies show that sustained engagement of the IL-1/Toll-like receptors not only propels malignant progression of *KRAS*-mutant pre-cancerous cells through amplifying tumor-intrinsic signaling and modulating extrinsic immune cells, but also contributes to treatment resistance of fully-transformed PDAC cells.

Obesity is an important clinical factor that drives engagement of the TIR receptors, mainly via increased intra-tumoral cytokines and possibly enhanced translocation of gut microbes. Various preclinical studies showed that obesity accelerates progression to PDAC in *KRAS^G12D^*-mutant GEMMs [28,72,73]. Mechanistically, adipocytes produce various inflammatory cytokines including IL-1α, IL-1β, TNF, and IL-6 [29,74], leading to production of more of these cytokines by PDAC cells and pancreatic stellate cells, ultimately drawing in inflammatory myeloid cells which aggravate intra-tumoral inflammation. In addition, obese mice have altered intestinal microbiomes [75] and possibly impaired gut barriers that permit translocation of gut microbiota into the pancreas, thereby accelerating progression of precancerous lesions to PDAC via engagement of the TLRs [76]. However, this latter hypothesis remains to be rigorously tested in relevant experimental models.

#### 4.1.3. IRAK4

Ligation of IL-1R and TLRs (except TLR3) recruits the adaptor protein MyD88 and the Interleukin-1 Receptor-Associated Kinases (IRAKs), which aggregate through interaction of their highly homologous N-terminal death domains to form a complex known as the Myddosome [77]. Within the Myddosome, IRAK4 is activated by dimerization and trans-autophosphorylation [78], which then signals through TAK1 kinase to engage the downstream JNK, MAPK, NF-kB, and activator protein-1 (AP-1) cascades [79]. Therefore, IRAK4 and TAK1 are master signaling kinases that can be therapeutically targeted to significantly curb inflammatory signaling.

Immunohistochemistry (IHC) analysis of PDAC tumor samples showed a strong positive correlation between activated p65/NF-kB and IRAK4 staining [59]. Notably, patients whose PDAC samples showed activated IRAK4 (by positive phospho-T385 IHC staining) in the neoplastic epithelia, which account for two third of all cases, had significantly worse progression-free and overall survival [59]. These results support IRAK4 as the driving mechanism of the NF-kB pathway in PDAC. Another closely homologous IRAK isoform, IRAK1, was shown to serve as a pseudo-kinase as mutation of its kinase domain did not affect the NF-kB activity of PDAC cells. On the contrary, replacement of endogenous IRAK4 with a kinase-dead mutant, or treatment with an IRAK4 kinase inhibitor markedly diminished NF-kB activity, invasiveness, and chemo-resistance [40,59]. Mechanistically, KRAS oncoprotein utilizes the MEK-ERK cascade to drive IL-1β expression, which through an autocrine manner activates IRAK4, the IKK complexes, and subsequently the TPL2 kinase [39]. Furthermore, IL-1β secreted from PDAC cells can activate IRAK4 and NF-kB cascade in the surrounding CAFs and subvert them into producing type 1 collagen and more IL-1β to foster a fibrotic and inflammatory milieu [40].

When challenged with cytotoxic chemotherapy, PDAC cells dramatically upregulate expression of TLR9, a DNA-sensing TLR, which also engages IRAK4 and TPL2 to further enhance the pro-survival MEK-ERK and NF-kB cascades [39]. Therefore, PDAC cells adaptively utilize different upstream receptors to engage a suite of downstream inflammatory signaling cascades mediated via IRAK4.

#### 4.1.4. TAK1

TGFβ activated kinase 1 (TAK1, or MAP3K7) was firstly discovered in 1995 in a complementation-based screening of a MAPK signaling in yeast by Matsumoto et al. as a signal transducer of TGFβ and bone morphogenetic protein (BMP) [80]. Other inflammatory receptors including the tumor necrosis factor receptor (TNFR), IL-1R, TLR, TGFb receptor, T cell receptor (TCR), and B cell receptor (BCR) also signal through TAK1, making it one of the most critical kinases that controls various cellular processes [81]. Global deletion of *TAK1* in mice results in early embryonic death due to severe developmental defects, particularly vasculature maldevelopment [82,83]. In PDAC, TAK1 can be activated directly or indirectly by oncogenic KRAS. Oncogenic KRAS upregulates GSK-3α, which binds TAK1 and stabilizes its interaction with the TAB complex to promote both canonical and non-canonical NF-kB cascades [84]. Enzymatic inhibition of TAK1 using a kinase inhibitor LYTAK1 sensitizes PDAC cells to the cytotoxic chemotherapies in vitro and in xenograft models [85], indicating the TAK1 kinase inhibitor as a promising new class of therapeutic agent that warrants further development. Further, gene expression analysis showed TAK1 to also regulate the Hippo pathway through stabilizing YAP and TAZ proteins. Mechanistically, GSK-3a-induces stabilization of TAK1, which in turn fosters the complex formation between TRAF6 E3 ligase and YAP/YAZ. This results in K63- rather than K48-polyubiquitination of YAP/TAZ proteins and protects them from proteasomal degradation. In this scenario, the kinase activity of TAK1 is not required [86]. At baseline and following genotoxic stress, IL-1R or TLR9-driven IRAK4 requires TAK1 kinase activity to engage downstream pro-survival cascades [81], thereby making TAK1 a highly relevant kinase in PDAC.

#### 4.1.5. TPL2

Tumor progression locus 2 (TPL2, also known as MAP3K8 or COT) is a serine-threonine protein kinase that mediates the IL-1 receptor, TLR, and TNF-dependent MAPK and NF-κB activation [87,88]. *TPL2* mRNA consists of an internal start codon, which produces two TPL2 protein isoforms of 58kDa and 52kDa. Activity of the TPL2 protein is regulated by phosphorylation and proteasomal degradation. In absence of receptor stimulation, TPL2 is bound to the NF-κB1/p105 protein complexed with the A20-binding inhibitor of the NF-κB (ABIN)-2 protein. This binding keeps TPL2 inactive and stable. Lipopolysaccharide (LPS), IL-1β or TNFα stimulation results in the activation of IKKβ, which phosphorylates p105, resulting in its degradation to p50 and simultaneously the release of TPL2 protein [89,90]. TPL2 undergoes phosphorylation at Ser400 by IKKβ and at Thr290 by an unknown kinase to become fully activated [91,92,93]. Activated TPL2 directly phosphorylates MEK1/2 and p105, leading to ERK1/2 (effector of MEK1/2) and p50 NF-κB transcription factor activation respectively [94,95]. In addition, TPL2 has also been shown to phosphorylate RelA/p65 NF-κB subunit at its Ser276 residue, MKK4/SEK1 (proximal kinase of JNK), and MKK3/6 (proximal kinase of p38α) in fibroblasts and macrophages stimulated with TNF-α or LPS [88,96,97]. In PDAC cells, TPL2 is activated via a KRAS-MAPK driven IL-1β autocrine signaling loop that engages IL-1R, IRAK4, and IKKβ [39]. In this setting, inhibition of TPL2 suppresses MEK-ERK, p-105, and p65 NF-kB activation, leading to enhanced survival and chemo-resistance. Targeting TPL2 with a small molecule kinase inhibitor significantly improves the therapeutic efficacy of chemotherapy (FIRINOX) in orthotopic and subcutaneous PDAC models [39].

Besides phosphorylation, protein stabilization is another critical mechanism by which TPL2 is regulated. TPL2 was initially discovered as a C-terminus truncated form from Moloney murine leukemia-induced rodent lymphomas and mouse mammary tumor virus-induced mammary adenocarcinomas [98]. In both cases, provirus integration occurs in the last intron of the gene, resulting in a C-terminus truncated/altered protein in which the last 43 amino acids are replaced with 7 amino-acids encoded by the intron. Later it was found that the C-terminus of TPL2 protein contains a “degron” that mediates its proteosomal degradation, and therefore C-terminally-truncated TPL2 proteins are more stable and cause higher ERK1/2 activation compared with WT TPL2 [99]. Similarly, truncations and fusions of TPL2 have been identified in melanomas. For example, in spitzoid melanoma patients, clinical sequencing uncovered such alterations in TPL2, which were mutually exclusive with alterations in other known driver oncogenes such as BRAF, NRAS, NTRK1/3, and ALK [100]. Interestingly, like the truncated gene, all the fusion transcripts lacked the final TPL2 exon. Consistent with the degradation function of C-terminus, immuno-histochemistry on patient tumors revealed that the truncated and fusion TPL2 proteins were expressed at a higher level, associated with increased phosphorylated (activated) MEK1/2, and these tumors were more responsive to MEK inhibition. Recently, work from our group showed that TPL2 can also be stabilized by point-mutations. From screening five different recurrent point mutations (E188K, R397H, R442H, L444V, and R459W) from TCGA database, the E188K and R442H mutants were found to be highly potent in inducing MAPK-SRE and NF-kB reporter activities [39]. The E188K mutation is found in oligodendroglioma, colon, and urothelial carcinoma, whereas the R442H/C mutations are found in colon, ovarian, gastric, and rhabdoid tumors. These mutations render TPL2 resistant to proteasomal degradation and thus are more potent than wild-type TPL2 in driving p-ERK1/2, p-S6, p-c-JUN, p-JNK, and p-AKT levels. Although TPL2 truncations or mutations have not been discovered in PDAC, high TPL2 protein expression predicts poor overall survival [39]. This observation warrants a need to understand the molecular mechanism, including the E3 ligase, lysine residues on TPL2, and upstream events, which regulate TPL2 protein stability.

To date, a clinical grade TPL2 inhibitor is not available. The rationale to target TPL2 is multi-factorial. Overexpression, truncation, fusion, and point mutation all indicate that TPL2 is a driver oncogene, hyperactivating the canonical MAPK, NF-kB, as well as JNK and p38 cascades. Phospho-activation and overexpression of TPL2 have also been implicated in therapeutic resistance and poor outcomes in PDAC [39]. Overall, these multiple facets of TPL2 biology foreshadow its success as a therapeutic target in PDAC as well as other cancer types.

### 4.2. Tumor Necrosis Factor (TNF) Pathway

Tumor necrosis factor (TNF) is a multifunctional inflammatory cytokine that has both pro-tumorigenic and anti-tumorigenic roles in human cancers [101]. TNF binds to two different receptors, TNFRSF1A (TNFR1) and TNFRSF1B (TNFR2). TNFR1 is expressed ubiquitously in all cell types whereas TNFR2 expression is largely limited to immune cells. Engagement of TNFR1 results in both pro-death pathways mediated by the RIP kinases, as well as pro-survival pathways through TAK1, NF-κB, JNK, and p38-MAPK signaling pathways [102,103,104]. In PDAC TME, TNF is produced predominantly by immune cells such as the macrophages, and partly by PDAC cells [105]. Basal intratumoral TNF expression is required for PDAC development, as anti-TNF treatment retards inflammation and reduces intratumoral macrophage infiltration in mice [106]. In *p48-Cre;KRAS^G12D^* (KC) mice, basal RIP1- and RIP3-induced necroptosis of PDAC cells leads to secretion of CXCL1, which recruits suppressive myeloid cells into the TME to incapacitate T cells [107], providing the rationale for blocking the RIP kinases to suppress myeloid cell recruitment. Further, tumor-intrinsic NF-kB activity can be boosted by exogenous TNF treatment, leading to chemotherapy resistance [108]. Autocrine TNF signaling was also shown to promote metastasis, as anti-human TNF blocking antibodies infliximab and etanercept blocked metastasis in PDAC xenograft models [109]. Therefore, basal intratumoral TNF level is required maintain a delicate balance which on one hand sustain PDAC cell survival and on the other hand allow a low level of PDAC cell necroptosis and release of chemokines (CXCL1) to help foster an immunosuppressive TME. Unfortunately, a phase I/II clinical trial combining etanercept and gemcitabine for advanced PDAC patients, though shown to be safe, did not prolong progression-free or overall survival compared to historical data on gemcitabine alone [110]. A clinical trial with RIP kinase inhibitor (NCT03681951) was prematurely terminated.

As the name implies, TNF is an extremely potent cytokine that can induce tumor necrosis. Delivery of high-dose TNF locally into tumors can induce massive necroptosis and tumor regression. Isolated limb perfusion of TNF plus melphalan produced an 89% response rate (61% complete response) in patients with melanoma [111] and a 73% response rate (26% complete response) in patients with sarcoma [112]. Endoscopy or percutaneously-guided intratumoral delivery of TNFeradeBiologic, a replication-deficient adenoviral vector that expresses TNF upon chemoradiation, appear to be beneficial in enabling surgery and thus better outcomes in a subset of patients with locally advanced PDAC [113], but this technique is not feasible for the majority of PDAC patients who have systemic metastasis. In fact, systemic administration of recombinant TNF has proven to be unacceptably toxic [114,115]. Therefore, manipulating intratumoral or systemic levels of TNF for therapeutic purposes is practically challenging.

A tractable strategy to harness the pro-death effect of TNF is to preferentially target the pro-survival cascade downstream of TNF receptor signaling. To this end, TAK1 and its downstream p38-MK2 kinase axis are promising targets. Binding of TNF to TNFR1 results in RIP-dependent recruitment of TAK1, which cooperates with MEKK3 to activate the pro-survival NF-κB cascade [116]. While being a modulator in chemoresistance [85], the role of TAK1 in normal pancreas development and stepwise PDAC progression has not been reported. Conditional deletion of *TAK1* (by crossing *TAK1^flox/flox^* mice with constitutive *villin-Cre* or tamoxifen-inducible *villin-CreER^T2^*) in intestinal cells results in spontaneous and rapid (<2–3 days) development of severe ileitis and colitis characterized by high expression of tissue IL-1β, TNFα, and MIP2. Notably, global deletion of *TNFR1* in enterocyte-*TAK1* deleted mice only rescued early, not late intestinal inflammation, probably due to compensatory overexpression of IL-6 and CXCL2 [117]. These results suggest that TAK1 signaling is essential for maintaining intestinal cell survival and homeostasis in a TNF-dependent and independent manner. Similarly, conditional deletion of *TAK1* (by crossing *TAK1^flox/flox^* mice with constitutive *Albumin-Cre* mice) in hepatocytes also resulted in spontaneous massive liver inflammation, fibrosis, compensatory hepatocyte proliferation, and subsequently the development of hepatocellular carcinoma [118]. Mechanistically, loss of *TAK1* in hepatocytes results in enhanced secretion of TNFα, IL-1β, and IL-6 from the surrounding Kupffer cells. Through engaging TNFR1, high tissue TNFα causes massive death of *TAK1*-deleted hepatocytes. In support, these events could be attenuated by global deletion of *TNFR1.* In this study, the development of hepatocellular carcinoma in liver-specific *TAK1*-deleted mice resulted from compensatory hyper-proliferation *TAK1*-deleted hepatocytes and does not suggest TAK1 as having a tumor suppressor function [118]. Together, these two studies underscore the essential role of TAK1 in sustaining enterocytes and hepatocytes survival in a highly inflammatory TME rife with cytokines such as TNF, IL-1β, and IL-6. Therefore, we hypothesize that TAK1 may similarly protect *KRAS*-mutant pancreatic cells from inflammation-induced cell death through sustaining intrinsic pro-survival pathways, making it a practical therapeutic target. However, this hypothesis must be experimentally proven using the appropriate PDAC models such as the KPC mice.

Recent work from our lab showed that PDAC cells exposed to FOLFIRINOX markedly upregulate TNF secretion and autocrine TNFR1 signaling, which drives RIP-dependent cell death programs and also TAK1-dependent activation of the p38-MK2 kinase cascade. Interestingly, we found that targeting TNFR1 or RIP kinases impaired FOLFIRINOX induced PDAC cell death, which is counterproductive. However, selectively targeting the downstream TNFR effectors, p38 or MK2 kinase, significantly increased PDAC cell death by FOLFIRINOX treatment. This anti-tumor effect of MK2 inhibition was due to decreased phosphorylation of HSP27 and BECLIN1, which are effectors of MK2 that mediate survival and protective autophagy respectively. Importantly, addition of MK2 inhibitor to FOLFIRINOX not only markedly prolonged the survival of KPC mice, but also reduced FOLFIRINOX-induced intestinal toxicities. Our study demonstrated that while manipulating intra-tumoral or systemic TNF levels is practically challenging, the anti-tumor effect of TNF can still be harnessed by selectively targeting its downstream signaling kinases including TAK1 and MK2 in order to improve treatment efficacy (Grierson et al., Science Translational Medicine, in press).

### 4.3. Interleukin Receptor-JAK-STAT Pathway

Constitutive activation of the signal transducer and activator of transcription (STAT) transcription factors, specifically STAT3, are very common in PDAC and are associated with poor patient survival [119,120]. Activated STAT3 (by phosphorylation) is detected by IHC in PDAC cells, myeloid cells, and a-SMA CAFs. The STAT transcription factors are typically phospho-activated by the Janus (JAK) family kinases under the control of upstream cytokine and growth factor receptors [121,122]. Mounting evidence now shows that the role of the JAK-STAT pathway is highly dependent on tumor type and cell type [123]. Studies from GEMM showed that *STAT3* is required for *KRAS^G12D^*-induced acinar-ductal metaplasia and PanIN formation, at least in part through upregulating *MMP7* expression [36,124]. On the contrary, genetic ablation of tumor-intrinsic STAT3 promotes *KRAS^G12D^*-driven lung tumorigenesis in mice. In lung cancer cells, STAT3 functions as a tumor suppressor by sequestering NF-κB within the cytoplasm and preventing it from transcribing *IL-8.* Because IL-8 is critical in recruiting suppressive myeloid cells and promoting tumor vascularization, loss of *STAT3* results in enhanced IL-8 secretion and lung cancer progression [125]. These examples highlight the importance of studying the role of each inflammatory pathway in relevant cancer models and caution against extrapolating findings from one cancer type to another.

Cytokines, including the interleukins and interferons, are the major mechanism that activates the JAK-STAT cascade [126]. Comparative gene expression analysis between PDAC cells and public databases showed IL-6 as the major cytokine that drives the JAK-STAT3 cascade in PDAC [36]. In a *p48-Cre* driven, doxycycline-inducible *KRAS^G12D^* mouse model, IL-6 is dispensable for pancreatitis-induced PanIN initiation but is required for the proliferation and survival of PanIN cells and subsequent progression to PDAC [127]. Besides mediating STAT3 activation, deletion of *IL-6* also impedes activation of ERK1/2 in PanIN cells, though these cells express *KRAS^G12D^*. This result indicates that feedback cytokines are critical in promoting oncogenic KRAS-induced PDAC cell progression by amplifying KRAS effector signaling and recruiting additional inflammatory pathways [127].

In PDAC tumors, IL-6 can be produced by PDAC cells under the control of oncogenic *KRAS* [36,38], myeloid cells [35], and pancreatic satellite cells (PSCs) [127,128]. Analysis of 73 blood samples from patients with untreated metastatic PDAC showed that high circulatory IL-6 was associated with poorer overall survival [129]. Mechanistically, ligation of the IL-6 receptor activates a different pathway that promotes PDAC cell fitness. First, activation of JAK-dependent STAT3 mediates transcription of *MMP7*, which is required for histologic progression [124]. Second, activated STAT3 can bind with IQ motif-containing GTPase-activating protein 1 (IQGAP1), which activates the small GTPase CDC42 to promote formation of pre-migratory filopodia and subsequently PDAC cell migration [130]. Third, activated STAT3 can recruit DNMT1 to epigenetically silence suppressor of cytokine signaling 3 *(SOCS3)*, whose gene product (SOCS3) is a natural inhibitor of JAK2, thereby resulting in sustained JAK-STAT pathway activation [131]. STAT3 inhibitor, AZD1480, plus gemcitabine attenuates the in vivo expression of SPARC (Secreted protein acidic and cysteine rich), increases micro-vessel density, and enhances intratumoral drug delivery. Accordingly, AZD1480 plus gemcitabine modestly prolonged the median survival of KPC mice (median survival 60 days vs. 52 days in control mice) [120]. Likewise, treatment of mice with an anti-IL6 receptor blocking antibody potentiated the anti-tumor activity of gemcitabine [132].

### 4.4. Transforming Growth Factor-β (TGF-β) Pathway

The TGF-β family of cytokines (TGF-β1, TGF-β2, and TGF-β3) are pleiotropic cytokines which critically regulate multiple normal cellular and physiological processes such as tissue homeostasis, cellular adhesion, differentiation, proliferation, and survival [133]. TGF-β binds with TGF-β type II receptor which then recruits type I receptor to form a heterotetrameric complex on the cell membrane, leading to activation of SMAD-dependent and -independent pathways. In SMAD-dependent pathways, the activated TGF-βRI/RII receptors phosphorylate the SMAD2/3 proteins in association with SMAD4. These activated SMAD complexes are translocated to the nucleus and bind with SMAD-binding elements (SBEs) and regulate the transcriptional of TGF-β-dependent genes [134]. In SMAD-independent signaling, TGF-βRI can recruit TRAF6 to activate TAK1 in a receptor kinase-independent manner [135], resulting in activation of IKK-NF-kB and JNK/p38 MAPK cascades. In PDAC tumors, TGF-b is produced by PDAC cells [136], pancreatic stellate cells [137], and regulatory T cells [138]. Importantly, TGF-β is produced in a latent form, which requires proteolytic cleavage to become an active ligand. Studies from *KRAS^G12D^*-driven GEMM showed that expression of αvβ6, an integrin family member that activates TGF-β, is progressively increased in neoplastic epithelia during stepwise neoplastic progression [139], suggesting that autocrine TGF-β signaling is partly controlled by availability of active TGF-β ligand through αvβ6.

TGF-β signaling has both tumor suppressive and promoting roles in human cancers, and its role is highly dependent on stage, genetic background, and cancer type. Greater than 50% of human PDAC exhibit loss of SMAD4 expression [140,141]. In *PDX1-Cre; KRAS^G12D^* (KC) mice, concomitant deletion of TGFBR2 greatly accelerates progression to invasive PDAC with 100% penetrance [142]. Similarly, loss of one or both alleles of the *SMAD4* gene, which has no biological impact, cooperates with *KRAS^G12D^* to accelerate progression to PDAC in KC mice [143,144,145]. It is now clear that the TGFβ-SMAD pathway drives cell cycle arrest and apoptosis during early phases of PDAC tumorigenesis, explaining why *SMAD4* loss propels *KRAS^G12D^*-driven tumorigenesis. In PDAC with *SMAD4* loss, TGF-β signaling is channeled predominantly through TAK1 and other kinase cascades, whereas in PDAC with intact SMAD function, both SMAD-dependent and independent pathways are involved in the malignant phenotype of PDAC including epithelial-mesenchymal transition (EMT) and chemotherapy resistance [146]. Specifically, induction of SNAIL expression and EMT by TGFβ-SMAD pathway requires expression of oncogenic *KRAS* in PDAC cells [147].

To date, it remains controversial whether inhibiting TGFβ-R1 indiscriminately is detrimental. Studies in *SMAD4*-intact KC mice showed that anti-TGF-β or anti-αvβ6 antibodies accelerated progression to PanIN and PDAC [139]. Furthermore, inhibiting TGFβ-R2 has contradictory effects in PDAC cell line models: on one hand it limits tumor formation, but on the other hand it promotes metastasis [148,149]. Therefore, until more data are available, targeting TGFβ-R1 should probably be limited to patients with *SMAD4*-mutated PDAC.

### 4.5. Impact of Onco-Inflammatory Network in Tumor Metabolism

Oncogenic KRAS induces metabolic reprogramming to provide energy and macromolecules that support the rapid proliferation of PDAC cells [150,151]. KRAS oncoprotein is known to induce autophagy, glycolysis, glutaminolysis, and micro- and macro-pinocytosis [152,153,154]. The role of autophagy is of particular interest in recent years. During tumor initiation, autophagy is thought to be anti-tumorigenic by limiting inflammation and ROS production. Impaired autophagic degradation causes intracellular buildup of autophagy substrates p62/SQSTM1. p62 is a signaling adaptor that promotes activation of NF-κB and the antioxidant nuclear transcription factor 2 (NRF2). NRF2-mediates expression of MDM2, which acts through p53-dependent and -independent mechanisms to relieve cell cycle checkpoints that blocks acinar-to-ductal metaplasia [155]. Furthermore, enhanced expression of NRF2 is sustained and very common in up- in PDAC cell lines and tumor samples [156]. Expression of oncogenes such as *KRAS^G12D^*, *BRAF^V600E^*, and *MYC* enhances basal expression of NRF2, which engages antioxidant program to lower intracellular reactive oxygen species (ROS) and create a more reduced intracellular environment that is more conducive of tumorigenesis [157,158]. In addition, *KRAS*-induced ROS formation and mitochondrial injury is also attenuated by PIN1-mediated NRF2 expression [159], which maintains intracellular redox homeostasis. Similarly, by maintaining redox homeostasis, NRF2 promotes stress-granule formation and expression of GPX4, leading to enhanced resistance to gemcitabine [160].

While efficient autophagy blocks acinar-to-ductal metaplasia during initial tumorigenesis, in fully transformed PDAC cells, robust autophagy program is critical in scavenging biomaterials that are critical in supporting cellular repair, energy production, and survival [161]. Furthermore, enhanced basal level of autophagy causes the continuous degradation of major histocompatibility complex class I (MHC-I) proteins, leading to evasion of PDAC cells from immune-surveillance [162]. Treatment of PDAC cells with MAPK pathway inhibitors such as MEK and ERK inhibitors further augments protective autophagy, which, when inhibited, results in greater apoptosis [163,164]. The mechanism that drives autophagy following MAPK pathway inhibition remains unclear. Recent work from our lab showed that treatment of PDAC cells with chemotherapy upregulates NF-kB dependent secretion of TNF, which activates autophagy in an MK2-dependent manner (Grierson et al., Science Translational Medicine, in press). Whether this is the same mechanism that drives autophagy following MAPK inhibition is unclear. If so, MK2 inhibition may emerge as a therapeutic strategy to block autophagy in combination with MAPK pathway inhibitors.

## 5. Clinical Trials Targeting Inflammation in PDAC

Targeting the inflammatory pathways to potentiate treatment response is supported by numerous preclinical studies. In Table 1, we summarized a few seminal past and recent clinical trials based on the molecular targets discussed above. Overall, targeting inflammation using these strategies has not yet achieved clinical success, likely for a few reasons. First, several inflammatory pathways have been shown to promote the malignant behavior and treatment resistance of PDAC. Targeting a single pathway or ligand, while being able to show statistically significant preclinical efficacy in mice, will likely be rapidly compensated for by other inflammatory pathways. Therefore, the signaling hubs through which multiple signaling pathways converge, such as TAK1, may serve as the “bottlenecks” that when targeted, may have a higher chance of success in clinical trials. Second, in established PDAC, inflammatory pathways function to aggravate the behavior of PDAC cells, largely through synergizing with intrinsic oncogenic pathways and recruiting CAFs and immune cells. Therefore, future clinical trials should include agents that can effectively curb the key oncogenic pathway (such as KRAS pathway inhibitors) or have substantial potential to eliminate PDAC cells (such as FOLFIRINOX or Gemctabine/Abraxane). Third, pertaining to the previous point, the cytokine profile within the TME and hence reliance on each pathway may be substantially different when PDAC tumors are at basal state versus when treated with chemotherapy. Therefore, preclinical studies should utilize models and include treatment regimens that will be used in clinical trials. Fourth, feedback contribution of CAFs and immune cells to the inflammatory pathways in PDAC cells cannot be fully studied using PDAC cells alone. Heterotypic cultures that include these different cell types should be included. Fifth, while the PDAC GEMMs are currently the only and best in vivo models that allow study of cell-cell interaction and thus are indispensable for studying inflammation, they are genetically oversimplified (carrying one or two mutations), homogeneous, and could have fundamentally different biology from humans. For instance, a comprehensive RNAi screen showed that mouse and human cells utilize different sets of signaling proteins in the TLR pathways [165]. Mouse and human cells also utilize distinct RAS effectors for tumorigenesis [166]. So far, the PDAC GEMMs have been a poor predictor of clinical efficacy. While better models are being developed, it is crucial to include both human and murine models in preclinical research, and substantial preclinical efficacy such as tumor regression, prolonged stabilization, and survival benefit should serve as criteria for advancing any novel combination for clinical trials. Sixth, future clinical trials should be guided and/or informed by relevant blood and/or tissue biomarkers in order to inform on-target effect, mechanisms of sensitivity or resistance, and identification of patient subsets that benefit from the trial regimens.

## 6. Conclusions and Perspectives

PDAC is one of the most challenging cancers to treat effectively. The inflammatory stroma of PDAC is a major therapeutic barrier that has multifaceted mechanisms of driving treatment resistance. However, effective strategies to overcome this stromal-mediated resistance are not yet available, largely due to the underlying complex and dynamic biology in the tumor-stromal interaction that is not completely understood. The pro-tumorigenic role of the TME is best illuminated by patients: For patients who have undergone surgical resection of primary PDAC, adjuvant modified FOLFIRINOX, which is intended to eliminate micro metastases, is able to cure 20–25% of patients [174]. However, for patients with established, metastatic PDAC, FOLFIRINOX has a ~30–35% response rate and is not curative [2]. These results strongly suggest that the PDAC stroma is a barrier that impedes chemotherapy response and needs to be targeted. The recent advent of direct KRAS inhibitors raise high hopes that meaningful clinical efficacy or a “cure” is on the horizon for PDAC patients. Direct targeting of the KRAS oncoprotein is probably the most effective way to disrupt the onco-inflammatory network in PDAC. The discovery of cyclic peptide scaffolds that specifically bind KRAS^G12D^ oncoprotein is extremely exciting, but these compounds are still being optimized to allow cell permeability [11]. Mirati Therapeutics has developed a KRAS^G12D^ inhibitor, MRTX1133, but the mechanism of action and preclinical data remain unpublished. Revolution Medicines has developed a KRAS(ON) inhibitor that can target KRAS^G12D^ and KRAS^G12V^ oncoproteins and these have shown very promising results in preclinical models [12]. While these direct KRAS inhibitors hold high promise, we must also be aware that KRAS^G12C^ inhibitor sotorasib achieved an objective response in only 9% (1 out of 11 patients) of *KRAS^G12C^*-mutant PDAC patients [45]. This unexpectedly disappointing result further underscores the critical need to co-target other key signaling pathways that propel PDAC tumor cells. To this end, co-targeting the key signaling nodes within the onco-inflammatory network (Figure 2) represents a promising therapeutic strategy that can enhance clinical efficacy.

To date, the notion of the PDAC stroma as a mere physical barrier is no longer considered to be the major mechanism of treatment resistance [175,176]. Instead, the ECM and cells within the stroma have active signaling function towards PDAC cells. Our current review captured the key inflammatory pathways of TME which feedback to strengthen PDAC cell fitness. However, most of the literature cited utilized cell line or mouse models that are unperturbed. We should be aware that the inflammatory landscape within the TME is highly dynamic and will certainly change in response to treatment such as chemotherapy. Therefore, the TME function and signaling interaction with tumor cells should be studied using state-of-the-art preclinical models in the context of treatment regimens that are used in the clinic. Until then, we propose that targeting key signaling nodes which control multiple inflammatory cascades should be investigated as therapeutic strategies.

## Figures and Tables

**Table 1:** Summary of selected past and recent clinical trials targeting inflammatory pathways in PDAC.

Molecular Targets	NCT	Phase	Target Disease	Treatment Arm(s)	Status/Outcome
Anti-IL-1α	NCT04825288	I/II	2nd line advanced	XB2001, 5-FU/LV and liposomal irinotecan Placebo, 5-FU/LV and liposomal irinotecan	Recruiting
Anti-IL-1α	NCT03207724	I	2nd line advanced	Xilonix plus 5-FU/LV and liposomal irinotecan	Completed No results yet
Anti-IL-1β	NCT04581343	Ib	1st line advanced	Canakinumab, partalizumab (anti-PD1), nab-paclitaxel, and gemcitabine	Recruiting
Anti-IL1RAP	NCT04990037	Ib	1st line advanced	CAN04 and mFOLFIRINOX	Recruiting
IL-1R antagonist	NCT02550327	Pilot	1st line resectable	Anakinra, cisplatin, gemcitabine and nab-paclitaxel	Completed No results yet
Anti-TNF	NCT00060502	II	1st line advanced	Infliximab and gemcitabine	Completed No results yet
Anti-TNF	NCT00201838	I/II	1st line advanced	Etanercept and gemcitabine	Completed No benefit [110]
RIP1	NCT03681951	I	≥2nd line advanced	GSK3145095	Terminated
Anti-IL-6	NCT04191421	I/II	≥2nd line advanced	Siltuximab plus Spartalizumab (anti-PD1)	Recruiting
Anti-IL-6	NCT02767557	II	1st line advanced	Tocilizumab, nab-paclitaxel, and gemcitabine Nab-paclitaxel and gemcitabine	Active, not recruiting
JAK	NCT01423604	II	2nd line advanced	Ruxolitinib and capecitabine Placebo and capecitabine	Completed May benefit patients with CRP > 13 mg/L [167]
JAK	NCT02119663	III	2nd line advanced	Ruxolitinib and capecitabine Placebo and capecitabine	Completed No benefit [168]
STAT3	NCT02993731	III	1st line advanced	Napabucasin, nab-paclitaxel, and gemcitabine Nab-paclitaxel and gemcitabine	Completed No results yet
TGF-bR1	NCT01373164	Ib/II	1st line advanced	Galunisertib and gemcitabine Placebo and Gemcitabine	Completed. Potential benefit [169]
TGF-bR1	NCT02734160	Ib	≥2nd line advanced	Galunisertib and durvalumab	Completed Limited efficacy [170]
TGFb/SMAD	NCT03666832	Ib	2nd line advanced	TEW-7107 and FOLFOX	Recruiting
Anti-NF-kB	NCT00094445	II	Any	Curcumin	Completed Activity in a small subset of patients [171]
Anti-NF-kB	NCT03714555	II	1st line advanced	Disulfiram plus gemcitabine, nab-paclitaxel or FOLFIRINOX	Completed No results yet
Proteosome	NCT00052689	II	≥2nd line advanced	Bortezomib Bortezomib and gemcitabine	Completed No benefit [172]
COX2	NCT00068432	II	1st line advanced	Celecoxib and gemcitabine	Completed No benefit [173]
